# The mitotic regulator Hec1 is a critical modulator of prostate cancer through the long non-coding RNA BX647187 *in vitro*

**DOI:** 10.1042/BSR20150003

**Published:** 2015-11-26

**Authors:** Haifeng Wang, Xu Gao, Xin Lu, Yan Wang, Chunfei Ma, Zhenkai Shi, Feng Zhu, Biming He, Chuanliang Xu, Yinghao Sun

**Affiliations:** * Department of Urology, Changhai Hospital, Second Military University, Shanghai, 20043, China

**Keywords:** apoptosis, bioinformatics analysis, cell cycle, human prostate cancer, Hec1, long non-coding RNA BX647187

## Abstract

Hec1 (highly expressed in cancer) is a member of a conserved Ndc80 (nuclear division cycle 80) complex that regulates mitotic processes. Its overexpression is seen in various tumours and is associated with cancer progression. However, its expression pattern and role inhuman prostate cancer (PCa) still not clear. The aim of our study is to investigate the expression and functional role of Hec1 in human PCa. Hec1 expression was measured in 10 pairs of PCa cancerous and non-cancerous tissue samples by quantitative real-time (qRT)-PCR. The effects of Hec1 on PCa cells were studied by RNAi approach. Apoptosis and cell cycle were analysed by flow cytometry. Cells viability was evaluated using cell counting Kit-8. Cyclin B1–Cdc2 (cell division cycle 2) activity was measured by ELISA assay. Long non-coding (Lnc)RNAs regulated by Hec1 were gained from bioinformatics analysis. The role of LncRNA BX647187, regulated by Hec1, was finally characterized in PCa cells by siRNA. Our results showed that Hec1 mRNA and protein were significantly overexpressed in Human PCa tissues and several PCa cell lines. Silencing Hec1 markedly suppressed proliferation, promoted apoptosis and induced cell-cycle arrest in G_2_/M-phase in PCa cells. Through bioinformatics analysis and knockdown Hec1 in PCa cells, we found LncRNA BX647187 was positively regulated by Hec1. We further demonstrated that suppression of BX647187 in PCa cells significantly reduced cell proliferation and promoted apoptosis. Thus, we conclude that Hec1 is consistently overexpressed in human PCa and Hec1 is closely linked with human PCa progression through the meditator LncRNA BX647187. Our studies may contribute to understand the molecular mechanism of PCa pathogenesis and clinical therapy.

## Abstract (precis)

The mitotic regulator Hec1 (highly expressed in cancer), is a member of a conserved Ndc80 (nuclear division cycle 80) complex that regulates mitotic processes. We find that Hec1 is consistently overexpressed in human prostate cancer and Hec1 is closely linked with human prostate cancer progression through the meditator LncRNA BX647187. Our studies may contribute to understand the molecular mechanism of PCa pathogenesis and clinical therapy.

## INTRODUCTION

Prostate cancer (PCa) is the most common malignancy and a common cause of cancer-related mortality for men all over the word. According to statistics, PCa affects more than 240000 men in the U.S.A. and increasing numbers of new cases are diagnosed each year [1]. PCa also is recognized as a major and increasing health problem in China. At present, despite the considerable advances in diagnosis and adjuvant therapy, the clinical outcome of PCa patients has not been improved markedly. Thus, pathogenesis, diagnosis and medical treatments of PCa still are substantial clinical challenges. Accordingly, there is an urgent necessity to improve the molecular characterization of PCa, in order to facilitate understanding of PCa pathogenesis and improvement on diagnostic and therapeutic efficiency.

Genomic instability is generally considered to play an essential role in the tumorigenic processes [2]. Chromosome segregation dysfunction in mitosis process is one of the reasons that lead to genomic instability. Hec1 (highly expressed in cancer), a kinetochore outer layer component and spindle check-point regulator, is a member of a conserved Ndc80 (nuclear division cycle 80) complex that regulates mitotic processes [3]. It is well study that Hec1 is essential for recruitment of mitotic check-point proteins to the kinetochore and ensuring faithful chromosome segregation. More recent work suggests overexpressed Hec1 correlate with various tumours formation, including gastric cancer, breast cancer, lung cancer, liver cancer [4–7]. Previous findings strongly suggested the role of Hec1 in tumour formation, nevertheless, its expression pattern and role in PCa still not clear [4–7].

In the recent years, Hec1 is also being studied as a novel anticancer target. Gurzov and Izquierdo [8] revealed Hec1 deletion in tumour cell lines induces mitotic abnormalities and cell apoptosis, which indicated Hec1 as a potential therapeutic strategy. Moreover, targeting Hec1 by RNAi or small molecules shows anti-tumour activity in animal models [9,10]. Therefore, Hec1 is considered as an excellent target for treating cancer clinically. Although extensive studies of Hec1 have been reported, the detailed molecular mechanism between Hec1 and cancers are only partially understood.

Long non-coding RNA (LncRNA) is a class of newfound noncoding RNAs that longer than 200 nts in length [11]. Previous studies show that LncRNAs play important roles in various cellular processes, such as cell proliferation, cell apoptosis, chromatin regulation and cell-cycle progression [11]. Recently, several LncRNAs have been shown to play important role in many tumour initiation and progression, such as lung cancer, hepatocellular cancer, gastric cancer, breast cancer, PCa and pancreas cancer [12–16]. Therefore, identifying the relationship between LncRNAs regulated by Hec1 and mitotic abnormalities may help to understand the function of Hec1 in pathogenesis of PCa.

In the present study, we sought to examine the expression and functional role of Hec1 in human PCa. We found that Hec1 is highly expressed in human PCa and several PCa cell lines. Then, we studied the impact of Hec1 deletion in PC3 cells via siRNA. In order to understand the mechanism of Hec1 regulates PCa, we further compare the LncRNA expression profile difference between tumour tissues and adjacent normal tissues. Finally, we also investigated the impact of altered LncRNA BX647187 levels, the most obvious change LncRNA, on the phenotypes of PC3 cells *in vitro*. To our knowledge, our findings for the first time revealed the involvement of Hec1 in human PCa.

## MATERIALS AND METHODS

### Tissue collection

All specimens were handled and made anonymous according to the ethical and legal standards. Paired tissue specimens (tumour and adjacent normal tissues) from 10 patients with PCa were obtained and histologically confirmed by a pathologist at Changhai Hospital, Second Military University from January 2010 to December 2010. All samples were derived from patients who had not received adjuvant treatment including chemotherapy or radiotherapy prior to the surgery in order to eliminate potential treatment-induced changes to gene expression profiles. After excision, tissue specimens were immediately frozen in liquid nitrogen for subsequent analysis.

### Reagents

Antibodies against caspase-3, caspase-8, caspase-9, Bax (Bcl-2 associated X protein), Bcl-2 (B cell lymphoma/lewkmia-2) and GAPDH (glyceraldehyde-3-phosphate dehydrogenase) were purchased from Cell Signaling technologies. Whereas, antibodies against Hec1, cyclin a2, cyclin b1, cyclin d1, cyclin E1, CDC2 and α-tublin were purchased from Abcam. Rabbit antibodies conjugated with horseradish peroxidase (HRP) and sheep anti-mouse-HRP were purchased from Zhong San Jin Qiao. All others chemical reagents were purchased from Sinopharm Chemical Reagent Co., Ltd.

### Cell culture

Human PCa cell lines LNCaP, PC3 and Du145 were obtained from the A.T.C.C. repository and human embryonic kidney cell line 293 T were purchased from Cell Bank of Chinese Academy of Sciences. Cells were cultured in Dulbecco's modified Eagle medium (DMEM; Gibco) supplemented with 10% FBS (Gibco), 100 units/ml penicillin and 100 μg/ml streptomycin and incubated at 37°C under 5% CO_2_.

### Quantitative real-time RT-PCR

Total RNA was extracted using Trizol reagents (Invitrogen) according to the manufacturer's instructions and diluted to 200 ng/ml. Then, quantitative real-time PCR (qRT-PCR) was performed using One Step SYBR® PrimeScript™ RT-PCR Kit II (TaKaRa) according to standard protocol. *GAPDH* gene was used as an internal control. The qRT-PCR amplification was performed as follows: 42°C for 5 min, 95°C for 10 s, followed by 40 cycles of 95°C for 5 s, 60°C for 20 s and 72°C for 15 s. PCR was followed by a melt curve analysis to determine the reaction specificity. The relative gene expression was calculated using 2^−△△Ct^ method. Primers used in qRT-PCR were as follows: Hec1: 5′-agaccttgggtatcctt-3′ (forward probe), 5′-tctttcatggcagtatgt-3′ (reverse probe); PSA: 5′-ttcctgcgtctgcttcct-3′ (forward probe), 5′-gtggctgacctgaaatacctg-3′ (reverse probe); GAPDH: 5′-ggaccaatacgaccaaatccg-3′ (forward probe), 5′agccacatcgctcagacac-3′ (reverse probe).

### Western blotting analysis

Cells were harvested and homogenized with cell lysis buffer (Beyotime). Then, the homogenates were centrifuged for 30 min at 4°C, 8000 ***g*** and the supernatants were collected as protein samples. Protein amounts were measured using BCA Protein Assay Kit (Beyotime). Equal amounts of protein samples were separated by denaturing SDS/PAGE (10% gel) and transferred on to PVDF membranes. Membranes were incubated in a 5% skim milk TBST blocking solution at room temperature (RT) for 1 h. And, membranes were incubated with agitation at 4°C overnight with specific primary antibodies against caspase-3 (1:1000), caspase-8 (1:2000), caspase-9 (1:1000), Bax (1:1500), Bcl-2 (1:1000), Hec1 (1:1000), cyclin A2 (1:2000), cyclin B1 (1:1000), cyclinD1 (1:5000), cyclinE1 (1:1500), CDC2 (1:2000) and p-CDC2 (1:1000). Then, membranes incubated by secondary antibodies (1:1000) conjugated with HRP at RT for 50 min. Finally, protein bands were visualized using an ECL western blotting detection system (GE Healthcare).

### siRNA

Cells were seeded (2×10^5^cells/well) in six-well plates. After incubation for 24 h, cells were transfected with siRNA targeting Hec1, BX647187 or negative control (si-Scramble) using Lipofectamine 2000 transfection reagent. The sequences of siRNAs were as follows: si-BX647187-1, 5′-agaagaagaagagagtgctt-tgcct-3′; si-BX647187-2, 5′-aagagagtgctttgcctgtagcatt-3′; si-BX647187-3, 5′-agagagtgctttgcctgtagcattt-3′; si-Scramble, 5′-agaagaagagagtgatttcggacct-3′; si-Hec1-1, 5′-gagtataaacaaacc-gacatctgaa-3′; si-Hec1-2, 5′-gatcccggaatagtcaacttggtat-3′; si-Hec1-3, 5′-ccggaatagtcaacttggtatattt-3′; si-Scramble, 5′-gagaa-ataaacagccctactatgaa-3′.

### Cell proliferation assay

The proliferation of PC3 cells was evaluated using cell counting Kit-8 (Beyotime, China) according to the manufacturer's instruction. Briefly, cells were transferred into a 96-well cell culture plate, with 200 μl suspension per well and grown overnight. After 24 h, the cells were transfected with siRNAs or si-Scramble. All groups were performed in triplicate. At 1, 2, 3, 4 and 5 day, 20 μl of CCK-8 (Cell Counting Kit-8) was added to each well respectively and then the plates were incubated for 2 h. Finally, absorbance was measured at 490 nm with a microplate reader (BioRad).

### Immunofluorescence

Cells were washed triple times with cold PBS and then fixed for 10 min with 4% paraformaldehyde dissolved in PBS at RT. Fixed cells were blocked for 1 h in 5% skim milk dissolved in PBS. Then cells were incubated with anti-α-tublin antibody overnight at 4°C in blocking solution and washed with PBST for three times. Following washing steps, cells were incubated with FITC dye-conjugated secondary antibodies for 1 h at RT. Finally, cells stained with 10 μg/ml DAPI (Beyotime) for 10 min. Images were observed and captured with a Nikon fluorescence microscope.

### Determination of apoptosis

The extent of apoptosis was determined by the flow cytometric measurement through AnnexinV-FITC apoptosis detection kit (Beyotime). Cells were treated as described above. After 4 days, cells were harvested and washed twice with cold PBS. Then, cells were stained in 1 ml of AnnexinV-binding buffer with 10 μl of propidium iodide (PI) solution and 5 μl of AnnexinV-FITC for 10 min at RT and analysed by flow cytometry.

### Cell cycle analysis

PC3 cells were seeded at a density of approximately 5×10^5^ in 100-mm plates and transfection with siRNAs for 48 h. Cell cycle was analysed by flow cytometry with PI staining using Cell Cycle Analysis Kit (Beyotime). Briefly, cells were harvested and washed with PBS and fixed with 70% ice-cold ethanol at 4°C overnight. Then cells were incubated in a PBS solution containing 10 mg/ml RNase and 1 mg/ml PI for 1 h at RT. Finally, the percentage of cells in different phases of the cell cycle was measured by flow cytometry (FACS Calibur, BD Biosciences). All samples were examined in triplicate.

### Kinase assays

Cyclin B1–Cdc2 (cell division cycle 2) activity was measured by ELISA assay using the MESACUP Cdc2–Cdk1 (cyclin-dependent kinase 1) Kinase Assay Kit (MBL). The kit is based on an ELISA that utilizes a synthetic peptide as a substrate for the Cdc2 kinases and a monoclonal antibody recognizing the phosphorylated form of the peptide. Firstly, total cell lysates were prepared for immunoprecipitation reaction and immunoprecipitated cyclin B1–Cdc2 complex were used for kinase assays. Then kinase activity was measured following the manufacturer instruction. Finally, the absorbance of each well at 492 nm was measured by a microplate reader (BioRad).

### Choice of differentially expressed LncRNA's list using heat map analysis

We obtained the microarray date from Gene Expression Omnibus (GEO, http://www.ncbi.nlm.nih.gov/geo/) and the GEO accession number is GSE3325. The date was generated using the genechip Affymetrix Human Genome U133 Plus 2.0 Array GPL570 (HG-U133_Plus_2), which completely coverage Human Genome U133 Set plus 6500 additional genes for analysis of over 47000 transcripts.

Observations with adjusted *P* ≥ 0.05 were removed and thus excluded from further analysis. The heat map of the 50 LncRNAs most obvious differences was created using a method of hierarchical clustering by GeneSpring GX, version 7.3 (Agilent Technologies).

Chosen LncRNAs were finally confirmed for altered transcription level using qRT-PCR between PCa tissue and PCa cell lines. Primers used in qRT-PCR were as follows: BX647187: 5′-agaggtgggagatgaggg-3′ (forward probe), 5′-cttctggcagcagtatgg-3′ (reverse probe). Other LncRNAs primer sequences are available upon request.

### Northern blot

Total RNA was prepared using Trizol reagents (Invitrogen) according to the manufacturer's instructions. Total RNA concentration was determined by measurement of absorbance at 260 nm. Northern blot was carried out according to the manufacturer's protocol (DIG Northern Starter Kit, Roche). Briefly, 20 μg of total RNA was denatured with formaldehyde and loaded into 1.2% formaldehyde-containing agarose gel for electrophoresis. Then, electrophoresed RNA was transferred to a nylon membrane and fixed with a UV cross-linker and finally probed with digoxigenin-labelled oligonucleotide probe of anti-BX647187 (5′-attgaagatgagatttgggt-3′) at a concentration of 10 pM, at 42°C overnight. Blots were processed using the Brightstar Detection Kit (Ambion) and developed on film. Northern blots hybridized with a 5S ribosomal RNA cDNA were used as controls.

### Statistical analysis

Data are reported as mean ± S.D. Statistical significance was determined using Double-sided Student's *t*test. Multiple groups were analysed using ANOVA. A *P*-value of less than 0.05 was considered to be significant.

## RESULTS

### Hec1 was highly expressed in prostate cancer tissues and cell lines

To evaluate the functional role of Hec1 in human PCa, the expression levels of Hec1 in 10 pairs of PCa tissue and adjacent non-tumour samples were determined by qRT-PCR assays. As shown in Figure 1(A), Hec1 mRNA level in PCa tissues were nearly 3.5-fold enhanced compared with those in adjacent non-tumour samples. Subsequently, we also detected the expression of Hec1 in three human PCa cell lines including LNCaP, PC3 and Du145 using qRT-PCR and western blot. Human embryonic kidney cell line 293T used as a negative control. High expression of Hec1 in PCa cell lines were also observed, especially in PC3 cells (Figures 1B and 1C). Prostate-specific antigen (PSA) is used as a positive control for PCa tissue.

**Figure 1:**
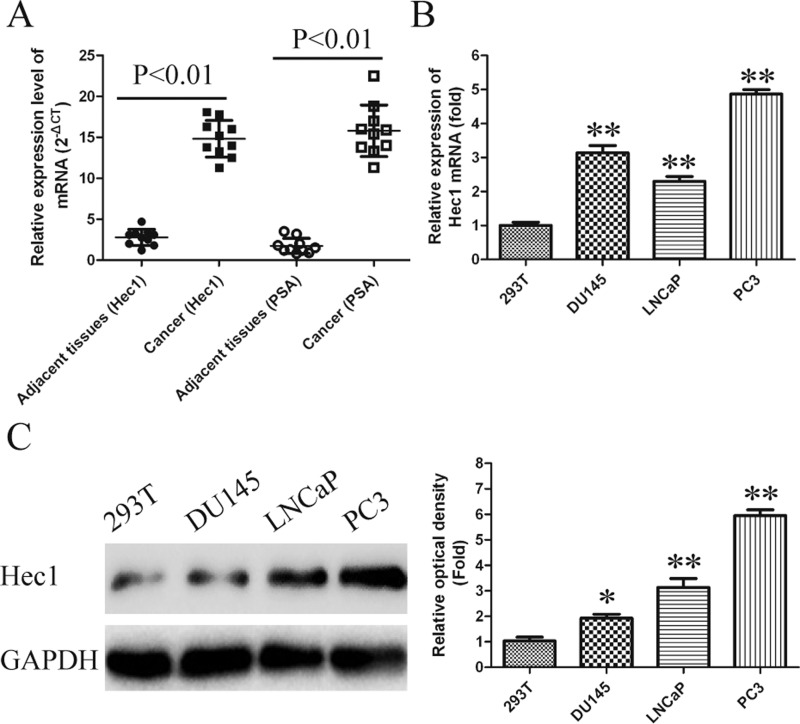
Increased expression of Hec1 in human PCa tissues and cell lines (**A**) mRNA levels of Hec1 were detected by qRT-PCR in 10 pairs of PCa tissues and adjacent non-cancer tissues. PSA is used as a positive control for PCa tissue. GAPDH gene served as an internal control. The relative gene expression was calculated using comparative cycle threshold method. (**B** and **C**) Protein and mRNA levels of Hec1 were detected by western blot assay and qRT-PCR in human PCa cell lines. GAPDH gene served as an internal control. The relative gene expression was calculated using 2^−△△Ct^ method. Date are expressed as means ± S.D. from three independent experiments and analysed by Student's *t*test.*P<0.05, **P<0.01 compared with adjacent non-cancer tissues. ^#^*P*<0.05, ^##^*P*<0.01 compared with non-tumour cell line 293T.

### Silencing of Hec1 inhibits cell growth and induces apoptosis in human PCa cell lines

In order to understand the functional role of Hec1 in human PCa, siRNA experiment was used to silence Hec1 in PC3 cells. As shown in Figure 2(A), the mRNA level of Hec1 in siRNAs transfected cells down to 0.6-fold, 0.2-fold and 0.4-flod respectively, comparing with scramble group. As shown in Figure 2(B), the down-regulated expression of Hec1 also observed using western blot. These results indicated that Hec1 was efficiently silenced in PC3 cells by siRNA–Hec1-2. Therefore siRNA–Hec1-2 is used for all subsequent Hec1 silencing experiments.

**Figure 2:**
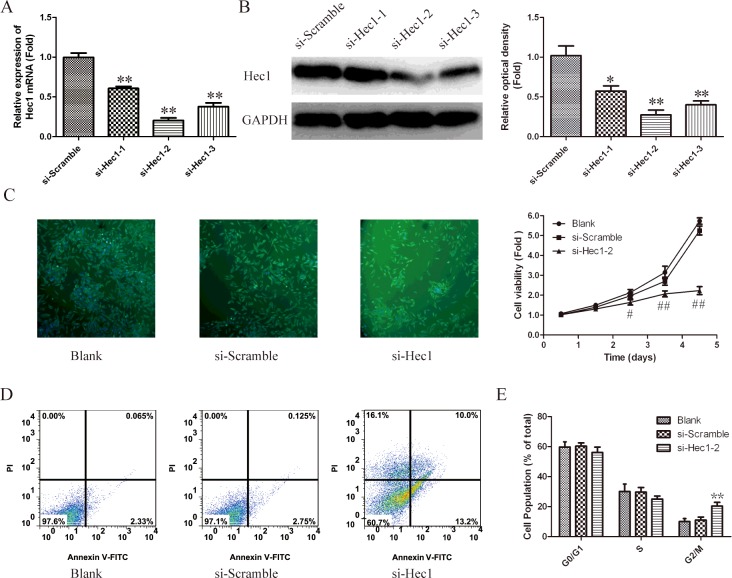
Effects of Hec1 knockdown on cell growth in human PCa cells (**A**) Silencing efficiency of si-Hec1-1, si-Hec1-2 and si-Hec1-3 were verified by qRT-PCR. *P<0.05, **P<0.01 compared with si-Scramble group.(**B**) Immunoblotting analysis of silencing efficiency of Hec1. GAPDH was used as an internal control. (**C**) Cell viability in PC3 cells which transfected with si-Hec1-2 was measured using immunofluorescence labelled with α-tublin antibody at 4 days and using CCK-8 kit. Values represent mean ± S.D. of three independent experiments. ^#^*P*<0.05, ^##^*P*<0.01 compared with blank group. (**D**) Flow cytometry analyse apoptosis of Hec-1 knockdown cells stained with AnnexinV-FITC and PI. (**E**) Cell cycle distribution was analysed by flow cytometry using PI staining. The percentage of cells in different phases was counted. Values represent mean ± S.D. of three independent experiments. *P<0.05, **P<0.01 compared with blank group.

To explore the effect of Hec1 in PCa cell growth, siRNA–Hec1-2 was transfected into PC3 cells and cells proliferation was measured by a CCK-8 assay. As shown in Figure 2(C), the proliferation rate of PC3 cells was remarkably reduced after siRNA–Hec1-2 transfection on the fourth and fifth day (*P*<0.01).

To investigate cell apoptosis caused by silencing of Hec1. PC3 cells apoptosis was measured by AnnexinV-FITC/PI double staining assay. As shown in Figure 2(D), the percentage of apoptotic cells in blank group and siRNA-scramble group is ~2.4% and 2.9% respectively. However, this value in siRNA–Hec1-2 group was increased to 23.2%. These results showed that Hec1 knockdown increased apoptotic rate of PC3 cells.

Then, the cell cycle distribution in PC3 cell lines was detected by flow cytometry. Compared with untreated cells and si-scramble transfected cells, Hec1 silenced cells were arrested in G_2_/M-phase (Figure 2E).

Taken together, these data indicated that Hec1 plays a critical role in human PCa and silencing of Hec1 inhibits cells growth and induces apoptosis in human PCa cells.

### LncRNA BX647187 was up-regulated in PCa cells

We used the microarray data GSE3325, submitted to the Gene Expression Omnibus (GEO) by Varambally at September 17, 2005 and last update at November 26, 2014, to analyse differential LncRNAs expression between PCa tissue and adjacent non-tumour samples. Firstly, hierarchical clustering analysis was used to compare differential LncRNA expression of the top 50 LncRNAs. The clustered heat map of 50 LncRNAs for GSE3325 is shown in Figure 3(A). Then, we further confirmed the selected six up-regulation of LncRNAs in PCa tissues and PCa cell lines through qRT-PCR. As shown in Figure 3(B), comparing with 293T cells, the expression of LncRNA BX647187 was significantly increased ~10-fold in PC3 cells, 8-fold in LNCap cells and 6.5-fold in Du145 cells. And all other five LncRNAs have a certain degree of up-regulation in PCa cell lines. In cancerous tissues, BX647187 expression was also at a level significantly higher than the average level of normal specimens (Figure 3C).

**Figure 3:**
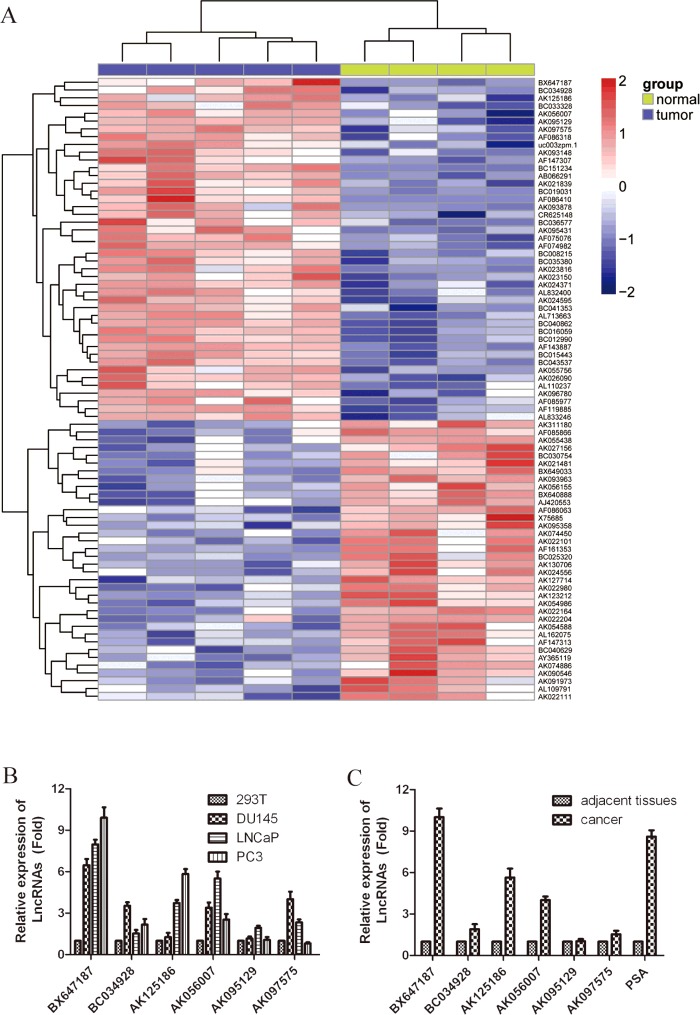
LncRNA expression profiles between PCa tissue and adjacent non-tumour samples (**A**) Heat map analysis of the LncRNAs expression of groups was created using a method of hierarchical clustering by GeneSpring GX, version 7.3. Rows: samples; columns: LncRNAs; colour key indicates LncRNA expression value, red: highest, blue: lowest. Microarray data obtained from Gene Expression Omnibus (GEO), GSE number is GSE3325. (B) Six biggest up-regulated LncRNAs in PCa tissue were further validated for altered transcription level using qRT-PCR. PSA is used as a positive control for PCa tissue. The relative amount of each LncRNAs was normalized to GAPDH. Dates in histograms are means ± S.D., *P<0.05, **P<0.01compared with normal group (*t*test).

### LncRNA BX647187 expression is regulated by Hec1

We next determined the relationship between Hec1 and LncRNAs. As shown in Figure 4(A), after transfected with siRNA–Hec1-2 in PC3 cells, the expression of LncRNA BX647187 was down-regulated by ~6.5-fold compared with blank or si-Scramble group. However, the expression of LncRNA AK125186 and BC033328 has no obvious change (Figures 4B and 4C). These data imply that BX647187 expression is positively regulated by Hec1.

**Figure 4:**
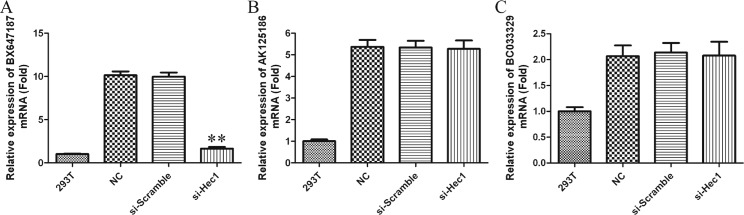
LncRNA BX647187 expression is positively regulated by Hec1 (**A**–**C**) BX647187, AK125186 and BC033328 mRNA levels were detected by qRT-PCR in Hec1 knockdown PC3 cells respectively. GAPDH gene served as an internal control. The relative gene expression was calculated using 2^−△△Ct^ method. Data are expressed as means ± S.D. from three independent experiments and analysed by Student's *t*test. *P<0.05, **P<0.01 compared with non-treated PC3 cells.

### LncRNA BX647187 regulates oncogenic phenotypes *in vitro*

We previously proved that silencing of Hec1 inhibits cell growth and induces apoptosis in human PCa cell lines. However, the molecular mechanism is undefined. In order to further verify whether the role of Hec1 in regulation of PCa cells was mediated via LncRNA BX647187, the expression of was knockdown via siRNA. The efficacy of siRNA is shown in Figure 5(A), more than 80% of the mRNA level was efficiently silenced in PC3 cells by siRNA-BX647187-1. As shown in Figure 5(B), declined transcript of LncRNA BX647187 is also proved by Northern Blot assay. As expected, BX647187 knockdown dramatically inhibited cell growth (Figure 5B). Since cell growth inhibition often due to cell-cycle arrest and increased apoptosis, we further analysed cell cycle and apoptosis by flow cytometry. As shown in Figure 5(C), the percentage of apoptotic cells was higher in siRNA-BX647187 transfected cells (15.2%) than that in si-Scramble transfected cells (5.1%) and parental cells (3.1%). To further determine the mechanism of BX647187 on apoptosis, the expression of caspase-3, caspase-8, caspase-9, Bax and Bcl-2 was analysed by western blot. As shown in Figure 5(D), the expression of caspase-3, caspase-9 and Bax were significantly increased and Bcl-2 was significantly decreased with treatment of siRNA-BX647187-1. But the expression of caspase-8 has no obvious change. Therefore, western blot results provided evidence that silencing BX647187 induced apoptosis through mitochondrial and caspases-dependent pathway.

**Figure 5:**
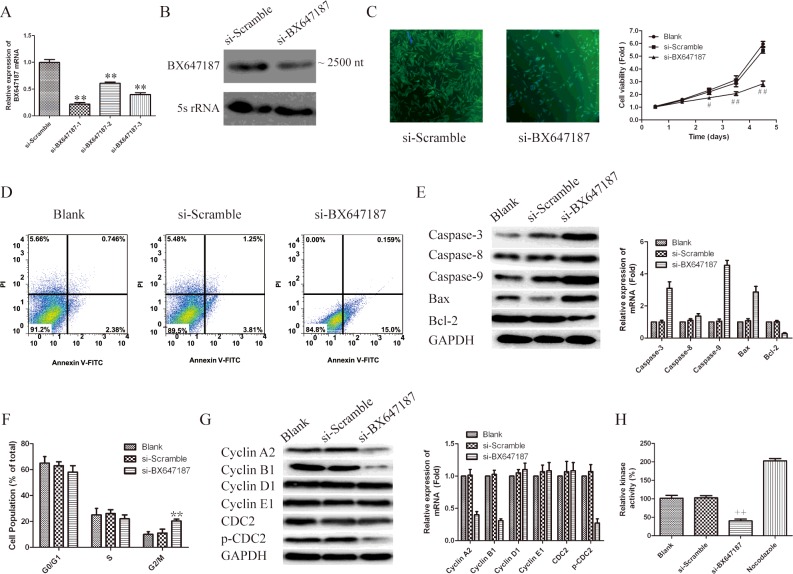
Knockdown BX647187 regulates oncogenic phenotypes of PC3 cells (**A**) Silencing efficiency of siRNAs was verified using qRT-PCR. *P<0.05, **P<0.01 compared with si-Scramble group. (**B**) The transcript of LncRNA BX647187 was detected by Northern blot. (**C**) Cell viability in BX647187 knockdown cells was measured using immunofluorescence labelled with α-tublin antibody at 4 days and using CCK-8 kit. Values represent mean ± S.D. of three independent experiments. ^#^*P*<0.05, ^##^*P*<0.01 compared with blank group. (**D**) Flow cytometry analyse apoptosis of BX647187 knockdown cells stained with AnnexinV-FITC and PI. (**E**) The expression of apoptosis relevant protein: caspase3, caspase8, caspase9, Bax and Bcl-2 were analysed through western blot. GAPDH was used as an internal control. (**F**) Cell cycle distribution was analysed by flow cytometry using PI staining. The percentage of cells in different phases was counted. Values represent mean ± S.D. of three independent experiments. *P<0.05, **P<0.01 compared with blank group. (**G**) BX647187 silenced PC3 cells and controls were subjected to western blot for determining the expression levels of cell cycle related proteins. GAPDH was used as an internal control. (**H**) Cyclin B1–Cdc2 activity determined by ELISA in cells nuclear extracts. ^+^*P*<0.05, ^++^*P*<0.01 compared with blank group.

After incubation for 48 h with siRNA-BX647187, 20% of PC3 cells were arrested in G_2_/M-phase, compared with only 10% of control groups located in G_2_/M-phase (Figure 5E). In order to further illuminate the G_2_/M-arrest observed, we examined cyclins A2, B1, D1, E1 as well as Cdc2 (cyclin-dependent kinases), p-Cdc2 (phosphorylated Cdc2, Tyr^15^). As shown in Figure 5(F), LncRNA BX647187 silencing led to a decrease in cyclins A2, B1 as well as a decrease in protein expression of p-Cdc2. However, the expression levels of cyclin D1, cyclin E1 and total Cdc2 remained unchanged. These data suggest that G_2_/M arrest is associated with activation of the cyclin B1–Cdc2. Finally, we also examined the kinase activity of cyclin B1–Cdc2 in siRNA-BX647187-treated cells. We found that the cyclin B1–Cdc2 kinase, an important regulator of cell cycle progression through G_2_/M, was dramatically inactivated in BX647187 knockdown PC3 cells (Figure 5G).

## DISCUSSION

In the present study, we report two novel findings: (i) Hec1 is consistently overexpressed in human PCa and cell lines and (ii) Hec1 is closely linked with human PCa development and progression through the meditator LncRNA BX647187 *in vitro*.

Previous studies suggest that Hec1 levels have a crucial role in many tumour formations [4–7]. A previous study found that high levels of Hec1 correlated with lung tumour grade and prognosis [6]. Many studies demonstrated that silencing Hec1 by construction of Hec1–shRNA (siRNA) in retroviruses, adenoviruses and adeno-associated viruses has potential for cancer gene therapy [8,17]. In addition, it has been also reported that using small molecule, which specifically disrupts the Hec1–Nek2 (NIMA-related kinase 2) mitotic pathway, retarded tumour growth in a nude mouse model bearing xenografts derived from human breast cancer cell line [9]. These reports indicated that Hec1 may serve as a therapeutic target for tumours. However, its expression and functions in human PCa is still unclear.

In the present study, we proved that Hec1 is overexpressed in human PCa and cell lines for the first time. This implies that Hec1 may also play a role in the development and progression of human PCa. Hence, we knockdown Hec1 in human PCa cell lines by siRNA. We found silencing Hec1 dramatically suppressed human PCa cell growth, induced cell apoptosis and arrested cell division at the G_2_/M-phase. Thus, we assumed Hec1 is critical in maintaining PCa cells growth.

There are several mechanisms that Hec1 be responsible for abnormal mitosis and tumour formation. A study by Deluca et al. [18] showed that the N terminus of Hec1 has key function of kinetochore microtubules (kMTs) dynamics and attachment stability and these functions depend on phosphorylation by Aurora B kinase. A relative study showed that HEC1 is required for other two check-point proteins Mad1 and Mad2 binding to kinetochores and play an essential role in chromosome segregation [19]. Hec1 depletion leads to defective mitotic check-point signalling and causes cell arrest at G_2_/M-phase. This arrest induces cell abnormal mitosis and apoptosis [20]. However, Hec1 may influence tumour through many routes.

LncRNAs have been found play widespread roles in many tumour cellular processes, including regulation cell proliferation, apoptosis and cell-cycle arrest in recent years. For example, Xu et al. [21] reported that LncRNA TUG1 is generally up-regulated in oesophageal squamous cell carcinoma (ESCC) and it promotes proliferation and migration of ESCC. Yin et al. [22] proved that LncRNA GAS5 affects cell proliferation and predicts a poor prognosis in patients with colorectal cancer. Additionally, it was also found high expression of LncRNA URHC (upregulated in hepatocellular carcinoma) can inhibit apoptosis through repressing expression of ZAK (sterile alpha motif and leucine zipper containing kinase AZK), a regulator of the ERK (extracellular regulated protein kinases)/MAPK (mitogen-activated protein kinase) pathway, in hepatocellular carcinoma [23]. Recently, some studies also showed that LncRNA participate cell cycle regulation through various routes [24–26]. Wang et al. [24] reported that LncRNA CCND1 (B-cell leukemia/lymphoma 1), which is transcribed from the upstream region of the cyclin D1 gene, participate in gene-specific repression of cyclin D1 and induce cell G_1_ arrest. LncRNA Gadd7 (growth-arrested DNA damage-inducible gene 7) was reported regulating CDK6 expression through negatively regulating CDK6 mRNA transcription indirectly [25]. Therefore, LncRNA Gadd7 controls cell G_1_/S-transition. In addition, it has also been reported that LncRNA–RoR (receptor tyrosine kinase-like orphan receptor) is a strong negative regulator of p53 gene, functions to control cell-cycle arrest and apoptosis [26]. Consequently, we propose the hypothesis that interaction between LncRNAs and Hec1 may play an important role in human PCa.

In the present study, we demonstrated that the expression level of LncRNA BX647187 is up-regulated in human PCa tissues and cell lines (Figures 3A and 3B) and is positively regulated by Hec1 (Figure 4A). Moreover, silencing BX647187 by siRNA could alter the phenotypes of PCa cells *in vitro*. We found inhibition of BX647187 showed lower cell viability and higher apoptosis compared with the control group in PC3 cell lines (Figures 5B and 5C). In addition, flow cytometric analysis showed BX647187 knockdown would lead to cells arrested in G_2_/M-phase (Figure 5E). Our data have identified an important role for BX647187 in human PCa development and progression.

To further elucidate the regulatory mechanism of BX647187 in G_2_/M cell-cycle arrest, proteins involved in some cycle regulators were analysed by immunoblotting. Our results indicated that silencing BX647187 markedly decreased the expression of cyclin B1 and the phosphorylated level of Cdc2 (Figure 5F). It has been widely accepted that cyclin B1–Cdc2 complex is required for cells transition from G_2_- to M-phase [27]. Then, the cyclin B1–Cdc2 kinase activity was also examined. We found cyclin B1–Cdc2 kinase dramatically inactivated in BX647187 knockdown cells. Thus, we conclude that LncRNA BX647187 regulates cell-cycle arrest at G_2_/M phase via inactivated cyclin B1–Cdc2 complex.

However, there are several limitations in our study that should be mentioned. For example, our sample size is relatively small. We do not distinguish whether overexpression of Hec1 was significantly correlated with clinicopathological parameters such as TNM (tumor node metastasis) stage, age and gender. Although we found LncRNA BX647187 expression is regulated by Hec1, the detailed regulation mechanisms between Hec1 and BX647187 are not understood. Besides, we detected Hec1 expression pattern and the relationship between Hec1, LncRNA BX647187 and pathophysiology in PCa cell lines. Nevertheless, these studies should be pursued by using appropriate animal models in future studies.

In conclusion, we found Hec1 is significantly high expressed in human PCa and several PCa cell lines for the first time. In addition, Hec1 plays a key role in PCa phenotypes via regulation of LncRNA BX647187. These findings would be helpful to understand the molecular mechanism of PCa pathogenesis and pathophysiology. We future analysis will focus on whether Hec1 and LncRNA BX647187 is a potential diagnostic even a therapeutic target for PCa.

## Abbreviations

Bax: Bcl-2 associated X protein
Bcl: Cdc2, cell division cycle 2
Bcl-2: B cell lymphoma/lewkmia-2
CCK-8: Cell Counting Kit-8
CDK: cyclin-dependent kinase
ESCC: oesophageal squamous cell carcinoma
Hec1: highly expressed in cancer
HRP: horseradish peroxidase
Lnc: long non-coding
Ndc80: nuclear division cycle 80
PCa: prostate cancer
PI: propidium iodide
PSA: prostate-specific antigen
qRT: quantitative real-time
RT: room temperature
